# Autophagy inhibition reinforces stemness together with exit from dormancy of polydisperse glioblastoma stem cells

**DOI:** 10.18632/aging.203362

**Published:** 2021-07-27

**Authors:** Aude Brunel, Sophie Hombourger, Elodie Barthout, Serge Battu, Donat Kögel, Patrick Antonietti, Elise Deluche, Sofiane Saada, Stéphanie Durand, Fabrice Lalloué, Marie-Odile Jauberteau, Gaëlle Begaud, Barbara Bessette, Mireille Verdier

**Affiliations:** 1 EA 3842 CAPTuR, GEIST Institute, University of Limoges, Limoges 87025, Cedex France; 2 Experimental Neurosurgery, Neuroscience Center, Goethe University Hospital, Frankfurt am Main D-60590, Germany; 3 German Cancer Consortium (D.K.T.K.), Partner Site Frankfurt, Frankfurt am Main D-60590, Germany; 4 Service d’Oncologie, CHU, Limoges 87025, France

**Keywords:** glioblastoma, cancer stem cells, plasticity, autophagy, SdFFF cell sorting

## Abstract

Therapeutic resistance and infiltrative capacities justify the aggressiveness of glioblastoma. This is due to cellular heterogeneity, especially the presence of stemness-related cells, i.e. Cancer Stem Cells (CSC). Previous studies focused on autophagy and its role in CSCs maintenance; these studies gave conflicting results as they reported either sustaining or disruptive effects. In the present work, we silenced two autophagy related genes -either Beclin1 or ATG5- by shRNA and we explored the ensuing consequences on CSCs markers’ expression and functionalities. Our results showed that the down regulation of autophagy led to enhancement in expression of CSCs markers, while proliferation and clonogenicity were boosted. Temozolomide (TMZ) treatment failed to induce apoptotic death in shBeclin1-transfected cells, contrary to control. We optimized the cellular subset analysis with the use of Sedimentation Field Flow Fractionation, a biological event monitoring- and cell sorting-dedicated technique. Fractograms of both shBeclin1 and shATG5 cells exhibited a shift of elution peak as compared with control cells, showing cellular dispersion and intrinsic sub-fraction modifications. The classical stemness fraction (i.e. F3) highlighted data obtained with the overall cellular population, exhibiting enhancement of stemness markers and escape from dormancy. Our results contributed to illustrate CSCs polydispersity and to show how these cells develop capacity to bypass autophagy inhibition, thanks to their acute adaptability and plasticity.

## INTRODUCTION

Glioblastoma (GB) is the most frequent and the most aggressive primary tumor of the central nervous system in adulthood. Despite the therapeutic care, including maximum safe resection surgery (when possible), followed by temozolomide (TMZ) chemotherapy associated with radiotherapy (corresponding to the Stupp protocol [1]), the prognosis remains dismal, and the median survival rate does not exceed 15 months [2]. The seriousness of this cancer is due to the combination of several characteristics. GBs display invasive properties, making them difficult to resect. They possess a high recurrence rate because of their therapeutic resistance, partly due to cellular heterogeneity, as emphasized by their previous designation as Glioblastoma "multiform" [2, 3]. Indeed, GBs, as reported for an increasing number of tumors, harbor a limited subset of cells, functionally related to stem cells, and currently named “Cancer Stem Cells” (CSCs) [4]. This cellular compartment is nowadays considered as a key determinant of GB aggressiveness, and is regarded as responsible for tumor relapse and therapy escape [5, 6]. Many studies have shown that this cell subset lies in the inner layer of the tumor mass, where pO_2_ reaches its lowest level [7]. Indeed, it is well known that hypoxic conditions promote stemness, in physiological as well as in tumoral contexts [8, 9]. According to the variable hypoxic gradient inside the tumor, it appears that GBs comprise different types of CSCs, reinforcing the intrinsic cellular dispersion [10]. It becomes clearer and clearer that the variability of this niche is linked to the variability of the tumoral cells, and especially CSCs, behavior [11, 12]. The plasticity of both niche and stem cells could explain why CSCs markers are so much variable [13], and justify using transcription factors such as SOX2, Oct4, BMI1 and NANOG in addition to classical stemness markers such as CD133 and CD44, in order to define this sub-population [14]. Even if they probably originate from a common ancestor, these various CSCs subsets exhibit different genetic and functional properties [14]. In addition, this sub-population is not static but could be now considered as either a flowing continuum in which multiple variants of stem cells can be found or as a non-hierarchical, reversible and adaptive cellular state [15].

The autophagic process is an evolutionary conserved degradation and recycling pathway, complementary to the ubiquitin-proteasome pathway. Autophagy involves the formation of autophagosome, a vesicle filled with fragments of cytoplasm and organelles, which is conveyed to the lysosome where degradation ultimately takes place. The building of the isolation membrane, which leads to the autophagosome, is a highly regulated process that involves a number of molecular actors, which operate in successive steps, i.e. nucleation, elongation, maturation and, finally, fusion with lysosome [16, 17]. Notably, Beclin 1 and ATG5 are essential components of respectively the nucleation and the elongation complexes, up-regulating autophagy. In addition to its basic role in cell renovation, autophagy allows the recycling of components (amino acids, fatty acids, etc.) and the production of metabolic energy, even in unfavorable conditions such as nutrient deprivation or hypoxia [18, 19]. Since this process fulfills a housekeeping function that contributes to cellular homeostasis, it appears rational that any dysfunction leads to some kind of pathology. In cancers, autophagy is considered as a double edge sensor, playing a role of tumor suppressor at the very initiation of the tumorigenesis, by elimination of damaged proteins and/or organelles and, at the same time, a pro-tumoral factor, since it provides some metabolic energy supply to cancer cells in response to metabolic stresses, once the tumor is developed [20, 21]. It is commonly thought that CSCs develop a protective autophagy, maintaining a sort of dormancy which ensures resistance to environmental stresses e.g. hypoxia and to either chemotherapy or radiotherapy [22–24]. The reverse hypothesis according which autophagy enhancement actually induces differentiation of CSCs and promotes therapy sensitivity, has also been supported by several studies [25, 26]. Then, we can legitimately wonder about the real nature of CSCs and their link to the autophagic process. According to Ryskalin et . [3], the question is still open to know which is the best therapeutic decision, induction or inhibition of autophagy, for GB treatment.

In order to focus on this special cellular sub-population, we developed in our laboratory the Sedimentation Field-Flow Fractionation (SdFFF) analytical approach, a label-free methodology which allows to enrich, isolate and characterize CSCs subpopulations from within a heterogeneous and polydisperse cell line. SdFFF is a sensitive, gentle, non-invasive, and tagless method that is particularly well-suited to sort stem cells and monitor biological events such as autophagy [27]. Although the SdFFF technique is related to high-performance liquid *chromatography (*HPLC), its major advantage lies in the absence of stationary phase. This one is replaced by a hollow channel, empty, delimited by 2 walls, one called accumulation wall, the other called depletion wall and in which a mobile phase flow. Thus, the analyzed samples - here, the cells - escape from interactions with a solid phase and are driven by flow streamlines of increasing speed from the walls to the center of the channel, characterizing their elution. Cell separation is dependent on the differential elution of species *via* the combined action of a parabolic profile generated by flowing a mobile phase through the channel and a multigravitational external field (generated by rotation of the channel) applied perpendicularly to the flow direction. Over the past decade, applications of SdFFF cell sorting have been developed in many fields, including oncology. A pioneering study of CSCs sorting from within a GB cell line [28] made it possible to sort other cancer cells [29] and to improve cell culture conditions ahead of cell sorting and CSCs detection [30]. Moreover, these studies allowed an opening towards the field of stem cell biology [31].

In a previous work, we showed that the autophagic and the neurotrophin pathways were both enhanced in GB cells, which were thus equipped to endure hypoxia [32]. Hypoxia, as well as autophagy and neurotrophins have been reported as boosters of the CSCs subset [33, 34]. However, the exact implication of this sub-population in the maintenance and dynamic cellular hierarchy, needs to be further explored. Thus, we decided to silence either autophagy or neurotrophin signaling by the means of shRNA and to monitor the ensuing consequences on the CSCs compartment. In order to optimize the analysis conditions and to refine our study with a technique specifically dedicated to cell sorting, we used SdFFF to isolate and distinguish these sub-populations. Our results showed that CSCs develop the capacity to bypass autophagy inhibition, by enhancement of proliferative capacities thanks to their functional plasticity.

## RESULTS

### Down regulation of autophagy awakened dormant GB CSCs

**Figure 1:**
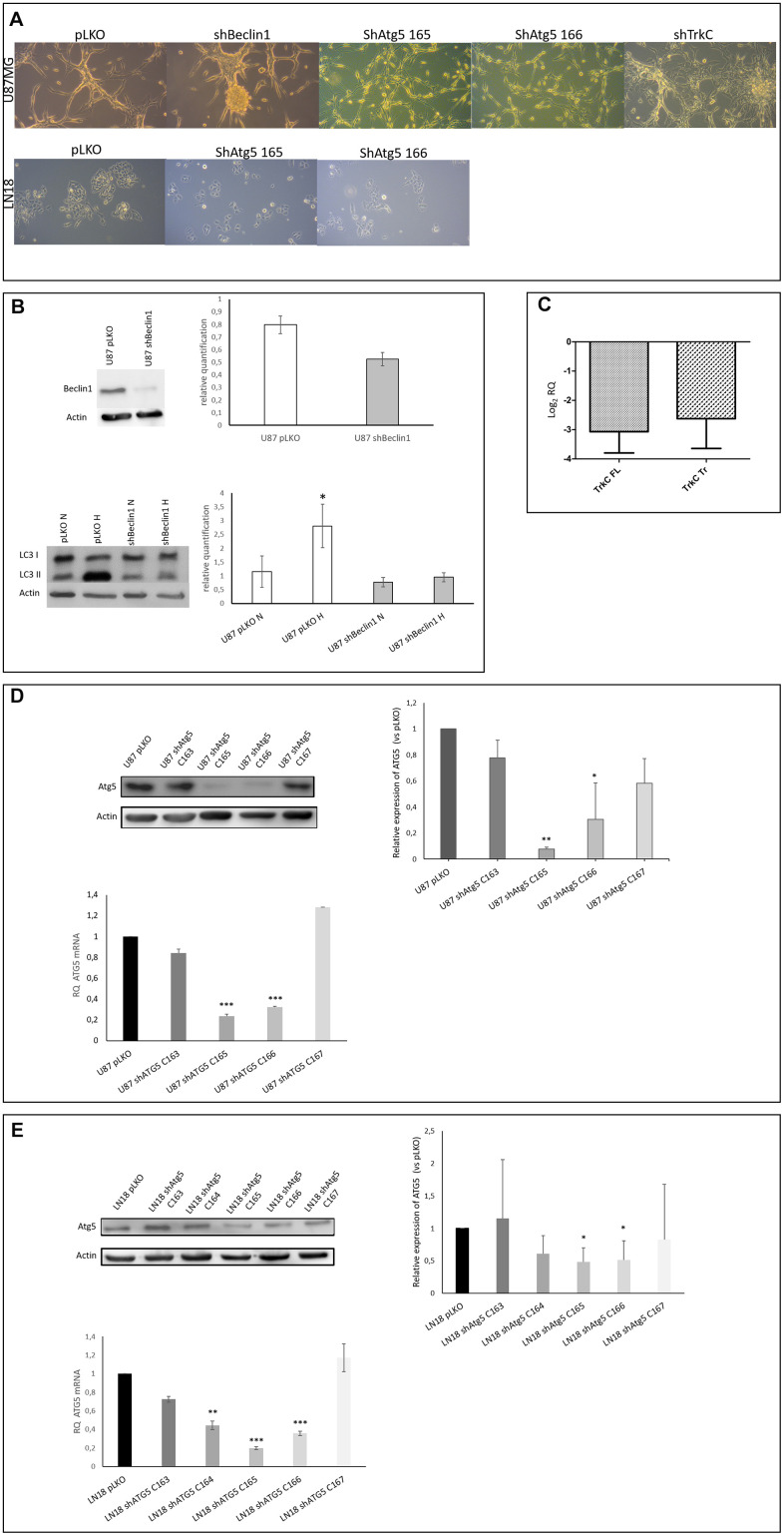
(**A**) Cell phenotype is modified in U87-MG but not in LN18 cells. U87-MG cells: U87shBeclin1 cells showed a high capacity to generate spheres as compared with U87pLKO cells (control cell line transfected with an empty vector). U87shATG5 cells (clones 165 and 166) appeared thinner and smaller than U87pLKO cells, without sphere formation. U87shTrkC cells appeared flattened, and larger than U87pLKO cells. LN18 cells: no difference was observed between LN18pLKO and LN18shATG5 cells, all cell lines presented attached cells, with a propensity to form clusters. (**B**) Reduced expression of Beclin1 leads to the inhibition of the autophagy process (N=3). Upper panel: Beclin1 protein expression is highly reduced in U87shBeclin1 cells showing a 60% of silencing in sh-cell line. Lower panel: LC3I to LC3II conversion (marker of functional autophagy) is evaluated by western blot analysis in U87pLKO and U87shBeclin1 cell lines when cultured in normoxic or hypoxic conditions. Increase of LC3II expression is observed in U87pLKO cells after hypoxic stress *p<0.05, whereas U87shBeclin1 cell line fails to exhibit such an increase. (**C**) Reduced expression of TrkC in U87shTrkC cells. TrkC expression was evaluated by RTqPCR. Biological activity of the TrkC receptor is due to two major isoforms of the TrkC protein after alternative splicing, named Full Length TrkC (145 kDa) and Truncated TrkC (95 KDa) (N=3). We verified that the TrkC-directed shRNA actually led to a drop in both TrkC mRNA levels (FL and Tr). In U87shTrKC both FL and Tr TrkC expressions, appear to decrease, with a log2 RQ value lower than -2.5 for each one of the transcripts (log2 RQ=0 corresponds to the expression level of TrkC in U87pLKO cells and was considered as a reference value). (**D**) Reduced expression of ATG5 in U87shATG5 cell lines (N=5). Upper panel: ATG5 protein expression was evaluated by western blot analysis in U87pLKO cells and in four U87shATG5 isolates. Clones U87shATG5-165 and -166 showed the strongest ATG5 expression decrease as compared with U87pLKO cells (**p<0.01 and *p<0.05 respectively). Bottom panel: the same evaluation was performed on ATG5 transcripts (RTqPCR) and gave similar results with the strongest decrease in ATG5 mRNA levels in clones U87shATG5-165 and -166 (*** p<0,001 for both clones). (**E**) Reduced expression of ATG5 in LN18shATG5 cell lines (N=5). Upper panel: ATG5 protein expression was evaluated by western blot analysis in LN18pLKO cell line and in four LN18shATG5 isolates. Clones LN18shATG5-165 and -166 showed the strongest ATG5 expression decrease as compared with LN18pLKO cells (*p<0.05 for both clones). Bottom panel: the same evaluation was performed on ATG5 transcripts (RTqPCR) in LN18 cell line and gave similar results with the strongest decrease in ATG5 mRNA levels in clones LN18shATG5-165 and -166 (*** p<0.001 for both clones).

In a previous work [32], we demonstrated the synergistic action of autophagy and the neurotrophin signaling pathway (i.e. TrkC) in GB cell survival, underscored by the exacerbated expression of these pathways in patient samples. In order to evaluate the involvement of autophagy and neurotrophin in CSCs maintenance and behavior and/or in the cellular ecology of glioblastoma, we constructed U87sh cell lines (U87shBeclin1, shATG5 and shTrkC) and checked the down-regulated expression of targeted proteins (Figure 1A–1D). Indeed, even if Beclin1 is described as being a determinant actor in autophagy initiation, we decided to check the data by silencing ATG5 a protein with a later onset of action in the process i.e. elongation of the isolation membrane.. Among the 4 shATG5 clones tested, the clone 165 led to a deeper silencing than the others (Figure 1D) and has been therefore chosen for further experiments. As shown in cell culture pictures (Figure 1A), the shBeclin1 cells were the most phenotypically modified cell line showing a higher propensity to develop spheres, a characteristic of immature cells. In comparison, the shATG5 cells appeared smaller than U87pLKO cells, without sphere formation.

**Figure 2:**
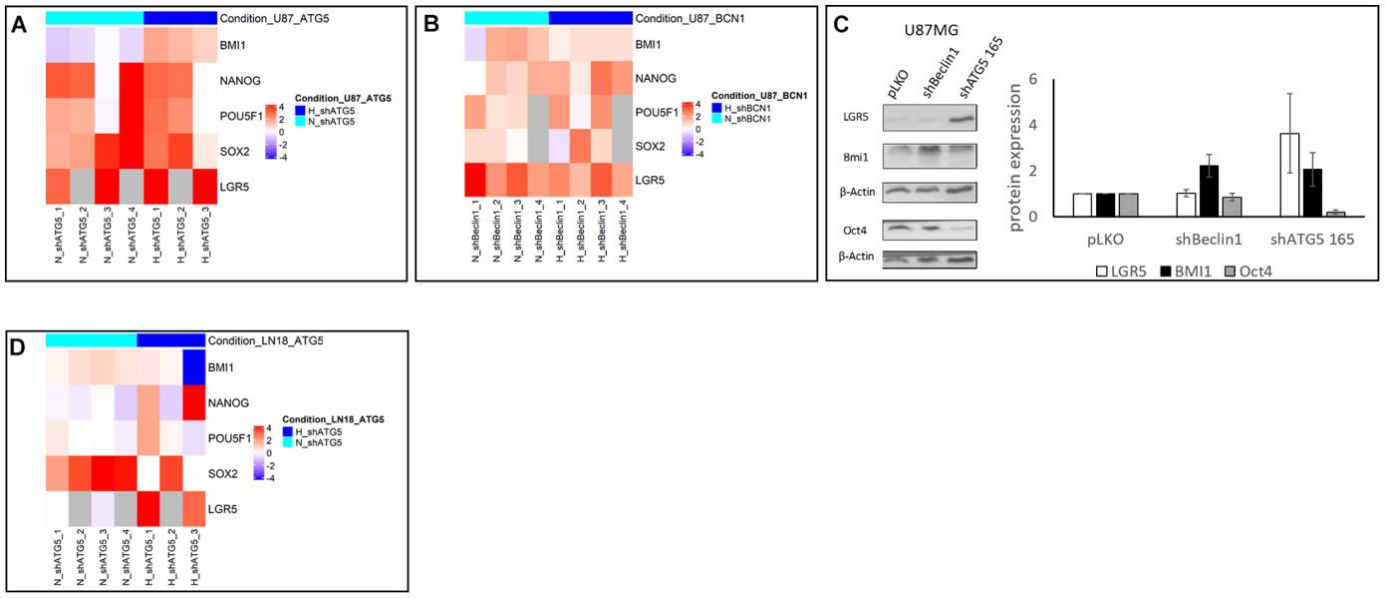
(**A**, **B**, **D**) Heatmap representation of stem cell markers in U87 and LN18 cell lines (N=3). Heatmap representation of stem cell markers expression level after extinction of Beclin 1 (BCN1) or ATG5 by RNA interference in normoxic (N, turquoise color) and hypoxic (H, blue color) conditions. Fold change has been calculated according to pLKO transfection with 2^-ΔΔCt^ method, and log2 transformed. Inhibition of BCN1 (**B**) or ATG5 (**A**, **D**) by shRNA transfection has been performed in U87 (**A**, **B**) and LN18 (**D**) cell lines. Fold change was indicated in logarithmic scale by a blue to red color gradient (under- to over-expression in shRNA versus pLKO conditions). Grey color corresponds to missing samples. Heatmaps were generated in R environment, using Complex Heatmap package. (**C**) proteomic expression of cancer stem cells markers and quantification (N=3). Cancer stem cells markers expression increases in two cellular model after autophagy inhibition (shBeclin1 and shATG5).

These observations led us to analyze the classical CSCs markers’ expression, i.e. BMI1, nanog, oct4, sox2 transcription factors and the WNT pathway co-receptor LGR5 (Figure 2). First of all, we checked that hypoxic conditions led to expected increase of stemness in U87 pLKO cell line. As shown in Supplementary Figure 1, expression of LGR5 and BMI1 cancer stemness markers was significantly enhanced when cells were cultured under hypoxic conditions p<0.05. Using RT-qPCR analysis, it appeared that U87shATG5 modified cell line expressed more intensely stem cell markers in comparison with U87pLKO either in normoxia or hypoxia, i.e. 1% O_2_ (Figure 2A). A similar enhancement was also observed when silencing another autophagy related gene, Beclin1 both in normoxia and hypoxia (Figure 2B). Western blot analysis of stemness markers, also exhibited an increased expression of BMI1 for both modified cell lines and of LGR5 for U87shATG5, whereas Oct4 expression failed to vary (Figure 2C).

To check and extend these results, we used another GB cell line i.e. LN18 (Figure 1A), in which autophagy was knocked down by the means of ATG5-targeting shRNA (Figure 1E). Similarly to U87 cell line, the clone 165 was more efficient for reduction of ATG5 expression and was chosen for further experiments. Concordant results at transcriptomic level were obtained, with significant enhancement of SOX2 expression in normoxic conditions and of SOX2, NANOG and POU5F1 in hypoxia (Figure 2D). This first set of data showed in two GB cell lines, that autophagy down regulation (by targeting two different genes) reinforces stemness phenotype.

**Figure 3:**
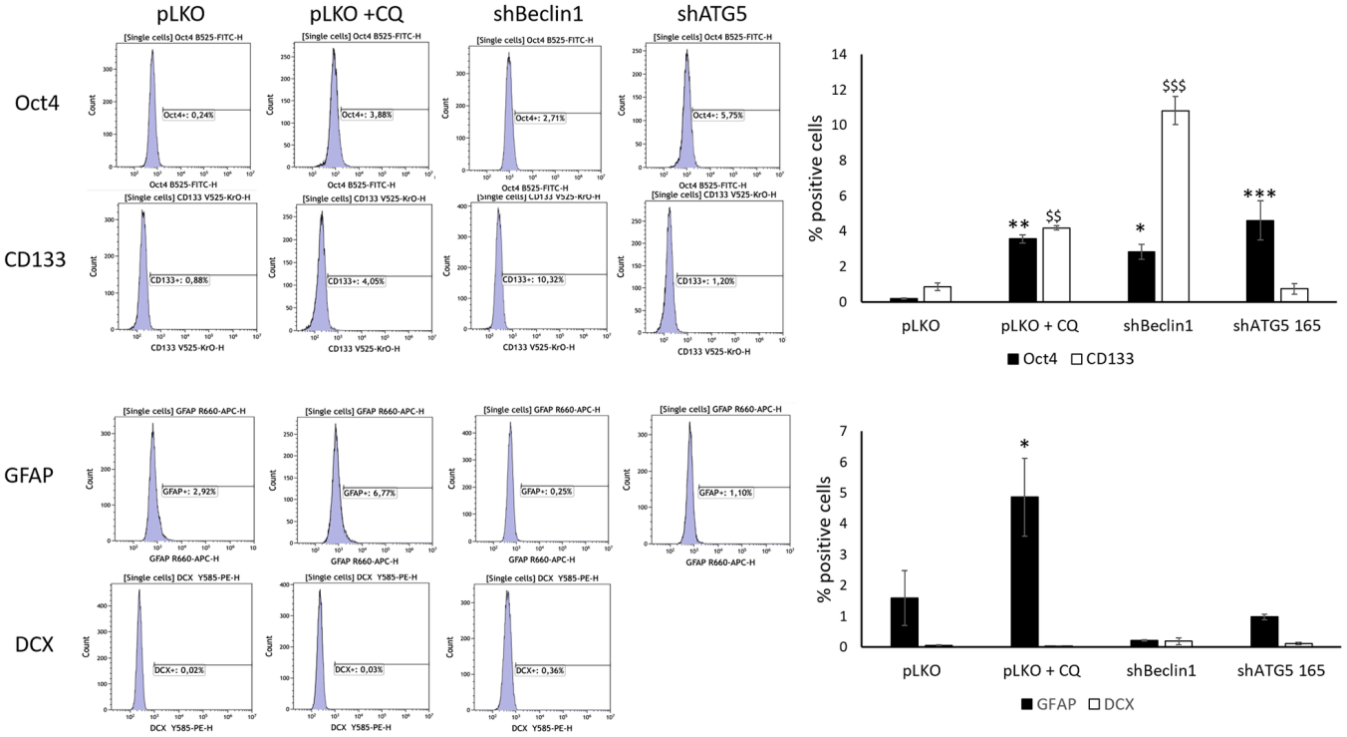
**Cancer stem cells and differentiation markers ‘expression were evaluated by flow cytometry.** (N=3). Chloroquine, Beclin1 and ATG5 protein inhibition significantly increase stem cell markers’ expression in U87-MG but do not significantly modify the expression of differentiation markers except for GFAP (* p<0.05). Cells were cultured for 96 h in normoxic or hypoxic conditions and were treated, or not, with 40 μM chloroquine (CQ) for 24 h, as previously described (32). In basal conditions, the percentage of pLKO cells expressing cancer stem cells markers is close to zero for both Oct4 and CD133. The two stem cells markers increased significantly compared to basal condition after inhibition of autophagy using Chloroquine (+ CQ, 4% ** and $$ p<0.01); in shBeclin1 cells (3% for Oct4 and 10% for CD133 * p<0.05 and $$$ p<0.001 respectively); in shATG5 cells (only for Oct4 *** p<0.001). Concerning differentiated markers no significant results are observed except for GFAP expression that increases after chloroquine treatment.

In order to confirm these results, we underwent a complementary study where specific differentiation markers of astrocytes (GFAP and Doublecortin) were assessed by the flow cytometry analysis in parallel to Oct4 and the classical stemness marker, CD133 (Figure 3 and Supplementary Figure 2). Furthermore, in addition to genetic autophagy silencing (shBeclin1 and shATG5), we used chloroquine (CQ) -treated U87pLKO cells, a well-known inhibitor of the autophagic flux. The enhancement of CSCs markers expression, Oct4 and CD133 was evidenced in the three conditions where autophagy was down-regulated (Figure 3, upper panel). In parallel, expression of the differentiation markers GFAP and DCX was impaired, especially in genetically modified cell lines (Figure 3, lower panel). To conclude, in response to this multi-way inhibition of the autophagic process (shBeclin1, shATG5 or CQ treatment) in two GB cell lines (U87 and LN18), the population of CSCs seemed to be potentialized rather than reduced.

**Figure 4:**
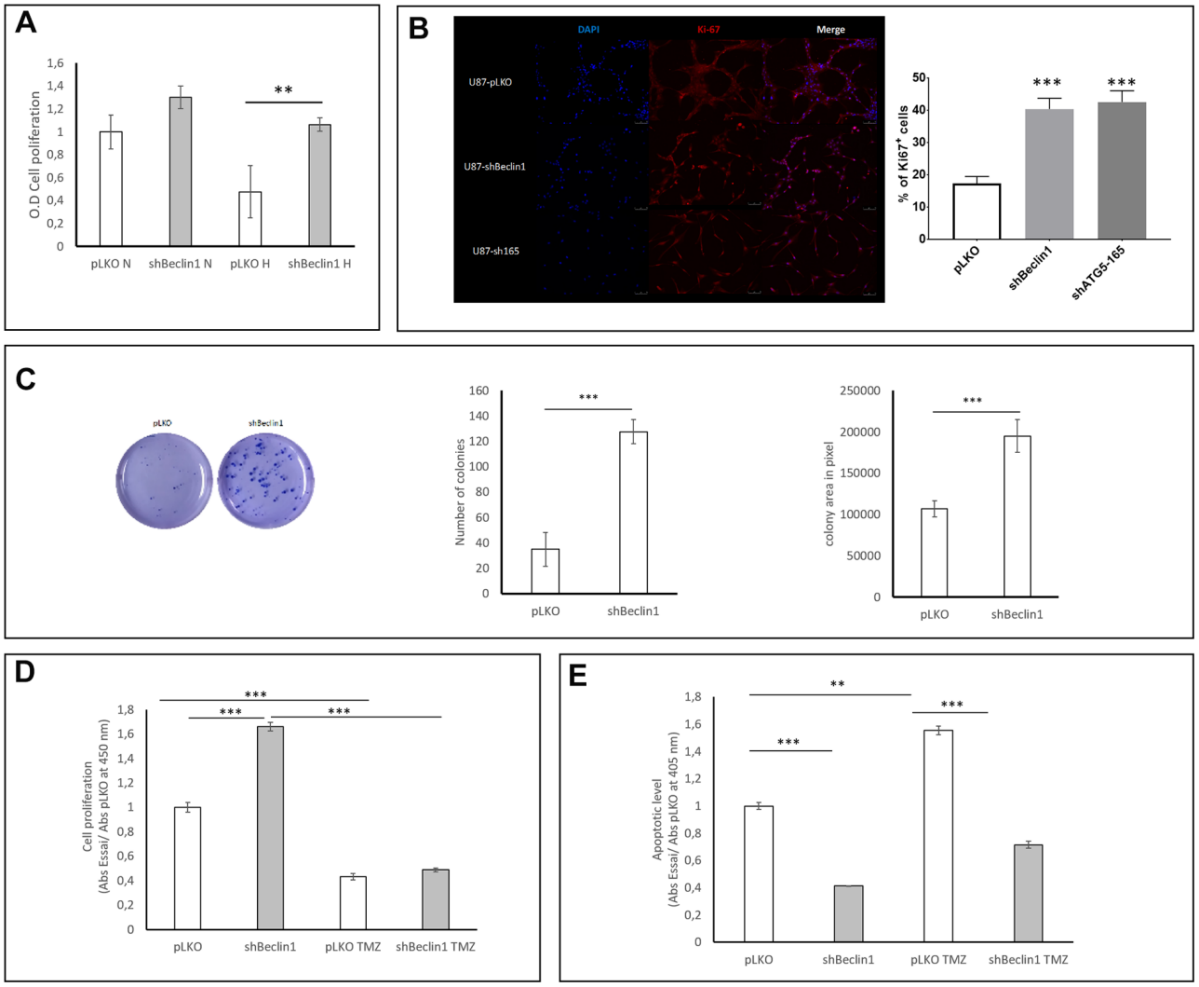
**Impact of Beclin1 extinction on glioma cell proliferation and clonogenicity.** (**A**) Cell proliferation in normoxic (N) and hypoxic (H) conditions. Cell proliferation was analyzed using BrdU incorporation (N=3). A slight increase in proliferation rate of U87hBeclin1 cells was observed, as compared with U87pLKO in normoxia; in hypoxic conditions a more significant difference was recorded **p<0.01. (**B**) Ki67 expression significantly increases in shBeclin1 and shATG5 cells compared to pLKO condition ***p<0,001 (**C**) Colony forming unit assay with U87shBeclin1. The clonogenic assay was performed as described in material and methods (N=3). There is a significant increase in number (left panel) and size (right panel) of U87shBeclin1 colonies as compared with those obtained with U87pLKO *** p<0.001. (**D**) Impact of TMZ on cell proliferation. In basal condition, after 144 h of cell culture and 3 h of BrdU incorporation, U87shBeclin1 cells proliferate more than U87pLKO (***p<0.001). TMZ treatment at 1.5 mM induces a very significant decrease in cell proliferation of the two cell lines (*** p<0.001) compared to non-treated cells. There was no difference in proliferative rates between U87pLKO and U87shBeclin1 cells when treated with TMZ (N=3). (**E**) Impact of TMZ on apoptosis. Apoptotic cell death was evaluated using the Elisa Cell death kit as described in material and methods. After 144 h of culture in basal condition, U87pLKO appears to be the most sensitive cell line to basal apoptosis compared to U87shBeclin1 (***p<0.001). TMZ treatment induces a significant increase in cell apoptosis of U87pLKO cell line (**p<0.01) whereas U87shBeclin1 cells appear to be resistant to TMZ-induced apoptosis (N=3).

Considering that the expression of stemness markers reflects a steady state of CSC, we explored the functional properties of the U87shBeclin1 cell line. We chose this modified cell line since when considering the morphology, the U87shBeclin1 was the only one to generate spheres, a characteristic of immature cells (Figure 1A). We analyzed cell proliferation with BrdU incorporation, at 96 h under normoxic and hypoxic (1% O_2_) conditions (Figure 4A) and at 144 h under normoxia only (Figure 4D). Both conditions increased cell proliferation of the U87shBeclin1 cell line, which reached a significant level under hypoxia at 96 h (**p<0.01 vs pLKO) and under normoxia at 144 h (***p<0.001). These results were confirmed by Ki67 staining in both shBeclin1 and shATG5 U87 modified cell lines (Figure 4B). This higher proliferation rate could not be attributed to an enhancement of MAP kinase or AKT pathways (Supplementary Figure 3). We also tested the clonogenic properties of U87shBeclin1 cells with CFU assay in agar matrix and evaluated colony numbers as well as colony areas, two parameters that characterize active CSCs. Down regulation of the autophagic process increased, at the same time, the number and the size of U87shBeclin1 colonies, in comparison with control (Figure 4C, ***p<0.001), hence corroborating proliferation rate enhancement. Since chemotherapeutic resistance is among the best known of the CSCs functional characteristics, we examined BrdU incorporation (cell proliferation) and apoptotic cell death after temozolomide (TMZ) treatment (48 h; 1.5 mM; Figure 4D, 4E). As expected, TMZ significantly decreased cell proliferation in both cell lines (control and U87shBeclin1). When we evaluated the apoptotic level by ELISA cell death after TMZ treatment, a significant increase of apoptosis was observed in U87pLKO (** p<0.01, Figure 4E) which was not the case of the U87shBeclin1 cell line. Indeed, this cell line exhibited a moderate apoptosis capability in whichever condition (***p<0.001).

As previously mentioned, our precedent work highlighted an exacerbated activation of the TrkC-NT3 axis in GB cell line and patients’ samples (32). That’s why, in complement to autophagy inhibition, the same analysis was done on U87shTrkC cells (Figure 1C). When the expression of the receptor was down regulated, we failed to observe neither variation in CSCs markers’ expression (Supplementary Figure 4A), nor in clonogenicity (Supplementary Figure 4C). Variations of proliferation (96 h, Supplementary Figure 4B and 144 h, Supplementary Figure 4D) and TMZ-induced apoptosis (Supplementary Figure 4D), seemed to be similar to the variations observed when autophagy was inhibited, although staying somewhat weaker.

Thus, since our initial goal was to affect CSC compartment, we chose to favor the autophagy inhibition approach in the subsequent study instead of knocking-down the TrkC signaling pathway. Indeed, inhibition of autophagy by the means of two different shRNAs showed an enhancement of the expression of CSCs markers and led to the forced awakening of CSCs, from the dormant state. So, we hypothesized that the different microstates of their CSCs were modified rather than a global modification of the overall population.

### SdFFF GB cell sorting allows to reveal CSCs heterogeneity

**Figure 5:**
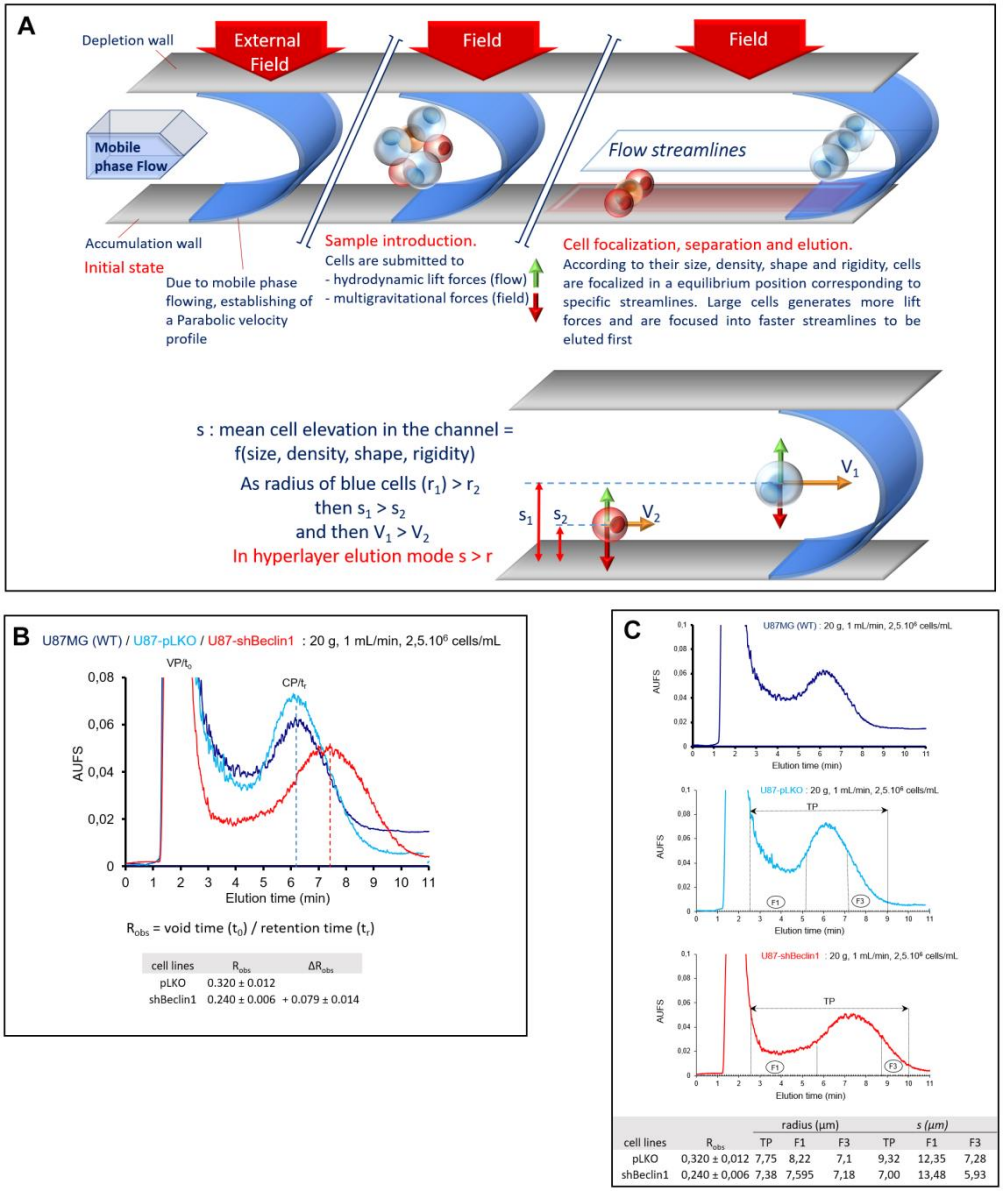
**Cell monitoring and cell sorting by SdFFF.** (**A**) A schematic representation of cell elution by sedimentation field-flow fractionation. Cells are eluted according to their biophysical properties (size, density, rigidity, deformability, nucleo-cytoplasmic ratio) under the biocompatible hyperlayer elution mode. Cell elution is characterized by the retention ratio: Robs = (retention time, tr)/void time, t0). Robs depends on cell elevation “s” in the channel thickness, ω. Larger and less dense cells are eluted first whereas smaller and denser ones are eluted later. (**B**) Comparison of representative fractograms. In each case, the classical two-peak profiles called fractograms were observed; the first peak corresponding to the void peak (VP, t0 unretained species) and the second one corresponding to the cell peak (CP, tr). Elution profiles (arbitrary absorbance units (AUFS) at 254 nm as a function of elution time) are similar for U87-MG (dark blue curve) and U87pLKO (light blue curve) cell lines. U87pLKO are used as control for SdFFF cell sorting. U87shBeclin1 fractogram (red curve) is around 1 minute right-shifted compared with U87pLKO. R_obs_ = t0 / tr (void time / retention time), calculated by the first moment method, are used for the measurement of the peak shift: ∆R_obs_ = R_obs pLKO_ - _Robs shBeclin1_ = + 0.079 ± 0.014 (N=4). (**C**) Representative fraction collections. Elution profiles are similar for U87-MG (upper panel) and U87pLKO (mid panel) cell lines but different for U87shBeclin1 (lower panel). Representative collected fractions TP and F are indicated on fractograms by horizontal lines bounded by arrows and vertical plane lines, respectively: TP: 2’30-9’00, F1: 2’30-5’10 and F3: 7’10-9’00 for U87pLKO; TP: 2’30-10’00, F1: 2’30-5’40 and F3: 8’45-10’00 for U87shBeclin1. Cells eluted in the fraction 1 (F1) are larger than those eluted in the last one (F3). "s" considered as the average cell elevation in the channel thickness is larger than the particle radius of U87pLKO cells, contrary to U87shBeclin1, especially cells collected in F3 shBeclin1(N=4).

In order to better characterize the CSCs population, we enriched the different fractions with the help of SdFFF. This tool allows cell separation depending on the combined action of a parabolic profile generated by the flowing mobile phase through the channel and a multigravitational external field (generated by rotation of the channel) applied perpendicularly (large red arrow on the depletion wall on Figure 5A) to the flow direction. Thus, larger and less dense cells generating more hydrodynamic lift forces within the mobile phase and not very sensitive to centrifugal force (blue cells on Figure 5A), will be eluted before smaller and denser cells (red cells, Figure 5A) generating less hydrodynamic forces and more sensitive to the multigravitational field. As for a number of other cell models [27, 29, 31], we found that under the elution conditions, U87 cells were eluted under the hyperlayer elution mode [28, 30] schematized in Figure 5A.

In each case, we observed the classical two-peak profile (Figure 5B); the first peak corresponds to the void peak (VP, t_0_/t_0_ = R_obs_ ≈ 1) containing unretained species (cell debris, proteins, cell aggregates…), and the second corresponds to the cell peak (CP, t_0_/t_r_ = R_obs_ < 1). These broad cell peaks (Figure 5B) correspond to very polydisperse populations, reflecting the heterogeneity of these GB cell lines containing a cell continuum, from fully differentiated cells to cancer stem cells [28, 30, 35].

As expected, elution profiles of the U87MG and U87pLKO cell lines are similar and characterized by quite identical retention ratios, R_obs_ = 0.319 ± 0.010 and R_obs_ = 0.320 ± 0.012 respectively. An important difference in elution profile was observed with U87shBeclin1 cells whose R_obs_ = 0.240 ± 0.006, corresponded to an important shift quantified by ∆R_obs_ = R_obs pLKO_ - R_obs shBeclin1_ = + 0.079 ± 0.014 (Figure 5B). This right shift led to a better resolution of the cell peak, well separated from the void volume, and associated with a flattened signal. In each one of the three fractograms, and according to the hyperlayer elution mode, the cells collected in fraction 1 (F1) are larger than those collected in the last fraction (F3). Interestingly, we observed that the “s” parameter of U87pLKO cells was larger than their average radius "r", while this is not the case of U87shBeclin1 cells, suggesting an increase of their retention time (≈ 1 min), without an important decrease in cell diameter. This is particularly true for fraction F3, as F3 U87shbeclin1 cells had a similar size to those of F3 U87pLKO (Figure 5C), demonstrating here that autophagy inhibition led to an important increase in cell density. This is usually associated with an increase in stemness properties. Once again, in order to check the strength of this peak shift specifically observed with the U87shBeclin1, we subjected U87shATG5 and CQ-treated U87pLKO cell lines to SdFFF (Supplementary Figure 5). In all the cell lines in which autophagy was down regulated, similar results were observed, the fractograms exhibited right-shifted with roughly similar ∆R_obs_ (Supplementary Figure 5). The same experiments have been conducted with the U87shTrkC cell line, but they failed to detect such differences (Supplementary Figure 6). The narrowing and the lowering of the second peak suggest a homogenization of the cell population, including the CSCs pool, which is highlighted by a left shift of the peak (∆ R_obs_ = R_obs pLKO_ - R_obs shTrKC_ = - 0.034 ± 0.014) (Supplementary Figure 6).

### SdFFF allows the isolation of a subset enriched in proliferative and clonogenic CSC

**Figure 6:**
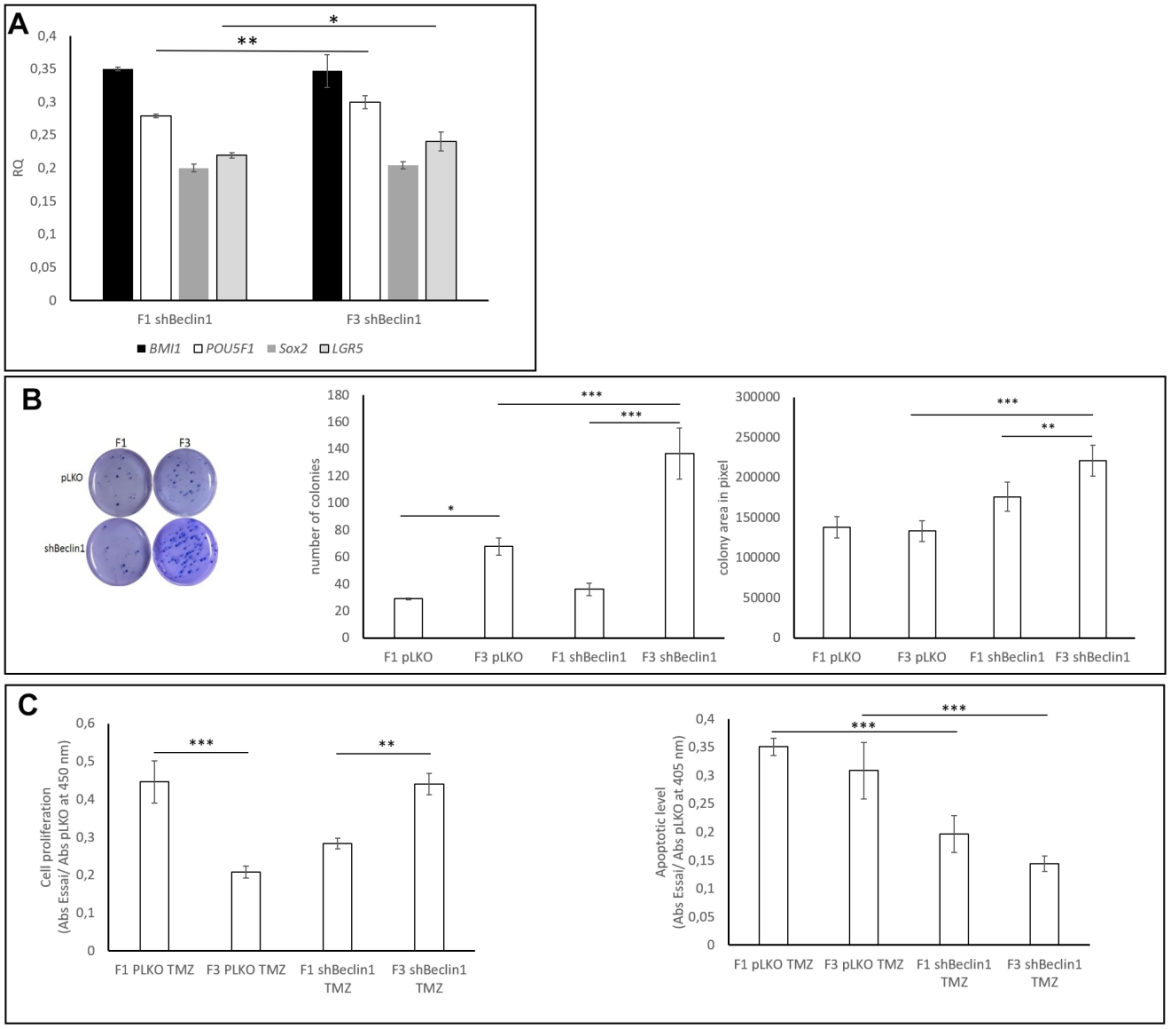
**Biological and functional characterization of SdFFF-sorted cells.** (**A**) Cancer stem cell markers expressions. CSCs markers’ expressions are compared to those observed in pLKO using RQ (2^-∆∆Ct^). *POU5F1* and *LGR5* gene expressions were significantly increased in F3 U87shBeclin1 cells compared with cells from F1 (respectively **p<0.01 and * p<0.05 for *POU5F1* and *LGR5*) (N=4). (**B**) Colony forming unit assay Left panel: The clonogenic assay was performed similarly to Figure 3B. Cells from the U87shBeclin1 F3 fraction appear significantly more clonogenic than cells from the U87pLKO F3 fraction. Mid panel: cells from the U87pLKO and U87shBeclin1 F3 fractions present a significant increase in colony number compared with colony number obtained from their F1 counterparts. * p<0.05 and ***p<0.001. In addition, colony number from U87shBeclin1 F3 is significantly higher than colony number from U87pLKO F3 ***p<0.001. Right panel: The size of U87shBeclin1 F3 colonies is significantly higher than the size of U87ShBeclin1 F1 colonies ** p<0.01. U87shBeclin1 F3 colony areas are significantly larger than areas of U87pLKO F3 colonies ***p<0.001. (**C**) TMZ impact on SdFFF-sorted sub-populations (N=3). Left panel: cell proliferation was examined after 48 h of 1.5 mM TMZ treatment using BrdU incorporation. U87pLKO cells from F3 fraction presented a significantly decreased proliferation rate compared with the corresponding F1 cells ***p<0.001. Cell proliferation of F3 U87shBeclin1 F3 cells was significantly increased as compared with corresponding F1 cells **p<0.01. Right panel: cell death was evaluated by Elisa cell death after 48 h of TMZ treatment at 1.5 mM. Apoptosis induced by TMZ in cells from F3 fractions was significantly more important in U87pLKO cells as compared with U87shBeclin1 cells *** p<0.001. Cell apoptosis in cells from F1 fractions was significantly more important in U87pLKO cells as compared with shBeclin1 cells ***p<0.001 (N=3).

We wondered whether the biophysical changes revealed by SdFFF analysis could help us to distinguish among sub-cellular populations, especially CSCs. As expected, and as already published [28], the F3 U87pLKO cell fraction exhibited more CSCs markers as compared with cells of the F1 fraction (data not shown). In the cell line in which autophagy was down-regulated (U87shBeclin1), analysis of CSCs markers confirmed a weak enrichment of the F3 fraction, based on enhancement of *LGR5* and *POU5F1* expressions (*p<0.05 and **p<0.01 respectively, Figure 6A). Expressions of the transcription factors *BMI1* and *SOX2* in the F1 and F3 fractions seemed to be fairly similar.

Comparison of clonogenic assays performed with cells of the F1 and F3 fractions obtained from U87shBeclin1 and U87pLKO cells showed a significant CSCs enrichment of F3 (U87shBeclin1) as compared with F3 (U87pLKO) (Figure 6B). Indeed, we observed that cells from F3 (U87shBeclin1) displayed increased colony size and number as compared with cells from F3 (U87pLKO) (***p<0.001, Figure 6B). Finally, we checked the impact of TMZ on these cellular fractions using BrdU incorporation and nucleosome quantification (Figure 6C). TMZ treatment had a specific impact on F1 and F3 cell proliferation in the two cell lines (Figure 6C left panel). F3 cells from U87pLKO appeared to be less proliferative than F1 cells (***p<0.001), whereas U87shBeclin1 F3 cells seemed to proliferate more than F1 cells (**p<0.01). Using “Genomics of Drug Sensitivity in Cancer” [36] (Supplementary Figure 7), U87-MG drug sensitivity demonstrated that temozolomide is still the gold standard of chemotherapy against glioblastoma. Nevertheless, in our experimental conditions (1.5 mM TMZ), pLKO appeared to be more prone to TMZ-induced apoptosis as compared with the shBeclin1 cell line, whatever the fraction considered (Figure 6C, right panel). As expected, the U87shbeclin F3 fraction, enriched in CSCs cells, exhibited the weakest apoptotic rate, when treated with TMZ (***p<0.001, F3 pLKO vs F3 shBeclin1, both TMZ-treated). Such a result showed that the CSCs enriched fraction issued from the lineage defective in autophagy summarized the data obtained with the whole population: expression of stemness markers was amplified, and CSCs came out from dormancy, being more proliferative and clonogenic. In addition, as described in Colorectal Cancer [29], we can distinguish between a quiescent and an activated state among the CSCs population.

When applied to U87shTrkC cell fractions, inhibition of TrkC signaling appeared to favor a less proliferating F1 sub-population and a less clonogenic F3 sub-population, and the two fractions were similarly resistant to temozolomide-induced apoptosis (data not shown).

## DISCUSSION

Aggressiveness of glioblastoma is partially due to i) - high proliferative potential and increased infiltrative capacities of the cells (associated with activation of growth factors belonging to the neurotrophin signaling pathway) and ii) - to the capability to strongly stimulate the autophagic process. We previously demonstrated a convergent and overlapping effect of autophagy and TrkC/NT-3 signaling, sustaining survival of glioblastoma cells subjected to hypoxia [32]. Based on these data, the initial goal of this study was to turn off either the autophagic process or the neurotrophin signaling (TrkC) in order to reduce GB cell survival rate and/or aggressiveness. We hypothesized that impairing these pathways would actually target and abolish the CSCs subpopulation.

Indeed, our results showed that down regulation of autophagy, either by the means of Beclin1- as well as ATG5-targeted shRNAs or CQ treatment, modified the behavior of CSCs. This observation was noticeable in two different cell lines i.e. U87MG and LN18. Especially, we used conventional stemness markers such as CD133, POU5F1, SOX2, BMI1, LGR5 and NANOG to identify the CSC compartment and we showed that their expressions were enhanced when autophagy was reduced. These results were mainly evidenced in hypoxic conditions, even if both cell lines and the two sh-targeted proteins led to responses of different intensities. Such variations could be explained by the different genetic backgrounds of U87 and LN18 cells, especially lack of amplified EGFR in U87MG and amplified EGFR in LN18, mutation in exon 8 of TP53 in LN18 and TP53 wild type in U87MG. Even if elevated expression of stemness transcription factors (SOX2, NANOG, FOXO3…) is usually associated with active autophagic process [37], a growing number of authors pinpoint the difficulty to base the identification of CSCs on the expression of static stem cell markers. Indeed, CSCs encompass a large variety of multipotent micro-states [3, 2], leading them from a very immature to a differentiated state, achieved in several stages. This cellular continuum has been erected as a model in the “*in situ*” GB tumoral mass, parallel to the O_2_ pressure [38, 7]. Such multi step progression could explain our results, being understood that even if autophagy was reduced, cells with stemness properties were still present in the culture. Furthermore, one cannot exclude a reversible transition between stem and non-stem cell states, supporting the tumoral heterogeneity and plasticity, as previously described [11].

Thus, the scientific community is presently more prone to take into account functional properties (clonogenicity, tumorogenicity, etc.) in order to identify such a plastic sub-population [15]. Indeed, we observed increased clonogenicity and proliferation of the U87shBeclin1 cell line, as compared with the U87pLKO counterpart. These results seem to indicate that knocking-down the autophagy-related protein Beclin1 abolished the quiescent status of stemness and favored the activated one, suggesting that these cells were coming out from dormancy. This transition corresponds to the SdFFF right shift of the U87shBeclin1 cell population, sign of an important increase in cell density. Similar results were obtained when silencing another autophagy gene expression involved in elongation of the phagophore, ATG5. Even if such a result tells us that the down-regulation of autophagy actors (Beclin1 and ATG5 in two cell lines) did not abrogate the CSCs subpopulation, it indicated that the behavior of GB cells was modified. Indeed, since autophagy provides the cells with energy and nutrients from their own constituents, it also contributes to their "dormancy", as well as their relative independence towards the microenvironment [37, 39]. Notably, it contributes to their adaptation to hypoxia, and, as a matter of fact, hypoxia and autophagy act together to maintain quiescence [19]. In this way, as our results showed, the cells begin to proliferate as soon as autophagy is no longer efficient. Furthermore, TMZ treatment of U87shBeclin1 cells only resulted in their reduced proliferation, without enhancement of apoptosis. Several studies (reviewed by Taylor et al. [40]), showed that TMZ led to autophagy exacerbation, resulting in cell death. It could explain why inhibited autophagy failed to induce apoptosis, which seems not to be the more preeminent TMZ-related cellular death. It is likely that the shBeclin1 modified cell line used another stemness-related cellular pathway to resist chemotherapy.

It is well known that GB tumors are heterogeneously composed, even inside the stemness compartment, as reported by the diversity of stem-associated gene signature [41]. Due to the pleiotropic role of autophagy in GB biology, it is no longer surprising that break-down of this process could affect and modify cellular behaviors [41]. Indeed, our data brought support to the “updated” concept of high plasticity of GB initiating cells [42, 43]. As mentioned by Hung et al. [44], the concept of CSCs state has evolved from a binary model (undifferentiated / differentiated) to a continuum that includes intermediate states, exhibiting partial stemness and differentiated phenotypes. Our data support such observation since, even when autophagy is breaking down by blocking the very initial step (i.e. Beclin1) or the elongation (i.e. ATG5), the CSC subpopulation remains, according to stemness markers expression such as CD133, Oct4, LGR5. However, functional properties (clonogenicity, proliferation, TMZ resistance) were modified especially in shBeclin1 cell line, leading to transient amplifying cells phenotype rather than quiescent CSC. We can't exclude that such phenotypical modification could also be linked to additional endocytic role of Beclin1 [41]. Finally, according to Prager et al. [2], we have to move from a hierarchic, binary model, where CSCs sit at the apex, towards a moving, heterogenous and dynamic stemness state. This reflection raises the question of the best way to study the transition between the different cellular compartments, given that expression of the CSCs markers probably lasts longer than the functional properties.

Facing the difficulty to study such a polydisperse minor cell population, we isolated cellular subpopulations thanks to SdFFF cell sorting. This biocompatible, label-free method was used to isolate CSCs-enriched sub-populations in order to better understand the behavior of the “silenced cell lines”. SdFFF has the great advantage of selecting sub-populations that can be characterized and whose functionality can be further studied. The geometry of the channel and their elution without contact with the walls protect cells from metabolic stresses that can signal their death, differentiation, autophagy... The counterpart is the dilution of the analytical cell sample limiting the number of cells in the sorted sub-populations forcing to increase the number of runs. Based on previous results of our group [28], we expected CSCs enrichment of the F3 fraction. Obtained data with sorted cells confirmed that the F3 fraction preferentially contained cells with characteristics of CSCs, as shown by the enhancement of *LGR5* and *POU5F1* expression, especially with the U87shBeclin1 cell line. Here again, the functional studies, and more particularly clonogenicity, indicate that the F3 fraction of shBeclin1 cell line seems to be more enriched in CSCs than the corresponding F3 fraction of the pLKO cells, whose clonogenic properties appeared more attenuated. When considering the TMZ effects, the F3 fraction showed a strong resistance to chemotherapy with the lowest rate of apoptosis. Considering these overall results, it appears that knocking-down autophagy by a shRNA directed against Beclin1 enhanced the CSCs subpopulation and affected the nature of these cells. Indeed, extinction of Beclin1 expression forced the F3 U87shBeclin1 sub-population to come out from the quiescent state and to express its proliferating and clonogenic abilities.

However, these modifications did not increase TMZ sensitivity, probably due to another compensatory mechanism. Based on our previous results, we explored the possible implication of the TrkC pathway in the regulation of GB CSCs behavior. Even if data associating stemness with neurotrophin are rather seldom, a previous study reported that neurotrophin signaling, especially *via* the TrkB and TrkC receptors, could sustain and enhance the GB stem cell compartment [33]. In our present study, shTrkC cells failed to display the expected cellular characteristics, dismissing implication of full-length TrkC signaling pathway in such a compensation. Nevertheless, this cell line seems to stay idle, exhibiting an "homogenization” of the cell population. Indeed, neither stemness markers’ expression nor clonogenicity were affected by TrkC silencing. The deprivation of neurotrophic proliferation signaling pathway results in the adaptation of their most resistant cellular pool, the CSCs, which ends-up with a quiescent metabolism. Another question we have to further explore deals with the metabolic modifications underlying the coming out from dormancy in the CSC sub-population (classically more prone to be quiescent). As highlighted by El Hout in a recent review [37], CSC maintenance takes advantage of mitophagy in order to reduce the mitochondrial mass, a process which favors glycolysis and quiescence. A recent study confirmed the enhancement of BNIP3 expression along with hypoxic conditions in GB patients derived cell culture [45]. Since this protein both inhibits apoptosis and favors mitophagy in GB, its variation of expression could be the missing link between CSC modified phenotype and autophagy manipulation. We still need a comprehensive view of what happens when stem cells are no longer maintained in the dormant state.

## CONCLUSIONS

The initial goal of our study was to turn off the CSCs compartment by impairment of the autophagic or the neurotrophic pathways. Even if this goal has not been fully reached (i.e. enhancement of stemness markers’ expression when autophagy was down regulated), this work supported the updated concept of “Cancer Stem Cells” heterogeneity. Indeed, we evidenced a CSC fraction consisting of cells endowed with proliferative properties. This cellular sub-population harbors a large number of multipotent micro-states [2], in which cellular adaptability depends on modifications of cellular processes such as autophagy. If we recognize that GB are such complex ecosystems, we may understand why an appropriate environment, with substantial variation in nutrients and oxygen availability, can influence this recycling mechanism, which in turn can modulate the stemness status, either positively (enhancement) or negatively (impairment). These updated concepts reinforce the question of the therapeutic potential of autophagy targeting, as underscored by Levine et al. [46].

## MATERIALS AND METHODS

### Cell culture and transfection

U87-MG and LN18 GB cell lines were purchased from American Type Culture Collection (ATCC). U87shBeclin1 and U87pLKO cell lines were provided by Dr Kögel and U87shATG5, U87shTrkC, LN18pLKO, LN18shATG5 cell lines were generated by transfection of U87-MG or LN18 cells with plasmids containing appropriate shRNAs (gift from Dr Priault, UMR CNRS 5095, Bordeaux). Four different clones were tested for ATG5 silencing (C163, C165, C166 and C167). Cell cultures were realized in Dulbecco’s Modified Eagle’s Medium (DMEM, Gibco, Life Technologies, Cergy-Pontoise, France), supplemented with 10% Fetal Bovine Serum (IDbio, Limoges, France) while transfected cells were grown in DMEM-FBS 10% containing 1 μg/mL puromycin (Fisher Scientific, Illkirch, France); both media contained GlutaMAX™. Cells were cultured under normoxia (5% CO_2_, 20% O_2_) or hypoxia (5% CO_2_, 1% O_2_), in a humidified incubator (Binder, Gennevilliers, France) for 96 h, seeded at a density of 10^4^ cells per cm^2^ and 2.5.10^6^ cells/mL suspensions are used for SdFFF cell sorting or monitoring.

Treatment with the chemotherapeutic agent temozolomide was performed at 1.5 μM upon 48 h after the 96 h culture or after cell sorting by SdFFF. Chloroquine treatments (40 μM, accordingly to previous study, 32) were only performed during 24 h after 96 h of culture, 2.5.10^6^ cells/mL suspensions were used for SdFFF monitoring.

### BrdU proliferation assay

Cell proliferation was tested with BrdU Cell Proliferation Assay Kit (Cell Signaling, Leiden, The Netherlands). Cells were seeded with 1,000 cells in 96 wells plates and cultured for 96 h or 144 h (96h + 48 h of TMZ treatment). Cells were then incubated with BrdU for 3 h at 37° C, then isolated, treated and incubated with the requested antibodies according to the manufacturer's instructions. Incubation with the horseradish peroxidase substrate 3,3',5,5'-tetramethylbenzidine (TMB), during 30 min was followed by addition of a Stop solution, and finally the reading of absorbance at 450 nm with a Multiskan FC Thermo Scientific microplate photometer (Thermofisher Scientific, Illkirch, France).

### Ki67 staining

U87-pLKO, shBeclin1 and shATG5-165 cell lines were grown on glass coverslips (14 mm diameter, Menzel-Glaser, VWR, France) in a 24-well plate at the density of 10 000 cells/cm^2^. After 72h of culture, cells were fixed in paraformaldehyde 4% for 15 minutes at RT and then washed with PBS. Saturation and permeabilization steps were performed using PBS-BSA3%-0.1% Triton X100 for 30 minutes at RT. The primary antibody anti-Ki67 (Santa Cruz, sc-7846) was diluted at the appropriate concentration in PBS-BSA3% and incubated overnight at 4° C with gentle agitation and in humid atmosphere. After several washes in PBS, the secondary antibody (Alexa Fluor 594, Invitrogen) was diluted in PBS-BSA3% and incubated for 2h at RT and protected from light. After PBS washes, DAPI (Sigma, D9564) staining was performed and mounting was realized using fluorescence mounting medium (Dako) before analysis under epifluorescence (Eclipse E800, Nikon, Champigny sur Marne, France).

### Cell death detection

This test is based on the quantitative sandwich-enzyme-immunoassay-principle (sandwich ELISA) using antibodies against nucleosomes (Cell Death Detection ELISA, Roche, Mannheim, Germany). Apoptosis is characterized by the splitting of the cell and the cleavage of DNA between nucleosomes, which is the most accessible part of the DNA, leading to the formation of mono or oligonucleosomes. Detection of nucleosomes allows the identification of apoptosis cell death. Cells were seeded at 10,000 cells per well and assay was realized according to manufacturer’s instructions. Absorbance was measured at 405 nm, using a Multiskan FC microplate photometer (Thermofisher Scientific, Illkirch, France).

### Colony formation unit assay

This test shows the ability of tumoral cells to form colonies, which is a property of stem cells. Cells (SdFFF-sorted or not) were seeded in wells of 6-wells plate (1,000 cells per well) in semi-solid medium constituted of 3 layers. The first layer contained 1 mL of mixed PBS containing 1% agar and culture medium (v/v : 1/1), the second layer contained 2 mL of PBS-agarose (0.7%) and culture medium (v/v : 1/1) added with 1 volume of cell suspension containing 1,000 cells per mL and the third layer consisted of 1.5 mL of culture medium. Cells were then incubated at 37° C and the medium was replaced every 3 days. After 21 days, the cells were fixed with 4% PFA for 15 min, stained using a 0.1% Crystal Violet solution for 20 min and washed several times with PBS to clearly distinguish every colony. Colony number and size were determined using ImageJ software (National Institute of Health, Bethesda, MD, USA). Experiments were performed in triplicate.

### Quantitative RT-qPCR

RNA extraction was performed on cell pellets using TRIzol® (Invitrogen, Illkirch, France). Pellets were dissolved in 500 μL of TRIzol®, then 100 μL of chloroform (Sigma Aldrich, Saint Quentin Fallavier, France) were added. Samples were shaken for 15 seconds and incubated at room temperature for 15 min., then centrifuged at 16,000 g for 15 min at 4° C. Upper aqueous phase was gently transferred in new tubes and the remaining phase was frozen at -20° C and kept for further protein extraction. RNAs were reverse-transcribed with the High Capacity cDNA Reverse Transcription kit, according to the manufacturer's instructions (Applied Biosystem, Illkirch, France). PCR runs were performed using Taqman® Fast Universal PCR Master Mix (Applied Biosystem, Fisher Scientific, Illkirch, France); gene expression was detected with StepONePlus® Real-Time PCR System (Applied Biosystems, Illkirch, France) and normalized to GAPDH. Results were expressed as Relative Quantification values: RQ= (2^-Δ∆Ct^).

### Western blotting and semi-quantification of proteins

Protein extraction has been performed with two techniques: RIPA for pellets of total cultures, or TRIzol® for pellets from SdFFF sorting.

RIPA: Cells were lysed for 30 minutes at 4° C with RIPA buffer (50 mM Tris-HCl pH 7.4, 1%NP-40, 0.5% Na-deoxycholate, 0.1% SDS, 150 mM NaCl, 2 mM EDTA and 50 mM NaF, supplemented, prior to use, with 1 mM PMSF). Cells were disrupted by sonication at 60 kHz, then centrifuged at 16,000 g for 20 min at 4° C. Protein concentration was measured with the Bradford protein assay (Sigma Aldrich, Darmstadt, Germany) and Bovine Serum Albumin standard, and absorbance was measured at 595 nm with a Multiskan FC photometer (Fisher Scientific, Illkirch, France).

TRIzol®: 150 μL of absolute ethanol were added to the remaining phase kept after RNA extraction and the samples were gently shaken, incubated for 2-3 min at room temperature and centrifuged 5 min at 2,000 g at 4° C. Proteins were precipitated with 400 μL of isopropanol, then the samples were incubated for 10 min at room temperature and centrifuged for 10 min at 12,000 g at 4° C. Supernatant was eliminated and pellets were washed with 95% ethanol containing 0.3 M guanidine hydrochloride. Pellets were then diluted in 1 mL of the washing solution, incubated for 20 min at room temperature and centrifuged at 7,500 g for 5 min at 4° C. Supernatant was removed and pellets were resuspended in 500 μl of absolute ethanol; samples were shaken, incubated at room temperature for 20 min and centrifuged at 7,500 g for 5 min at 4° C. Pellets were air dried for 10 min max and resuspended in 1X Laemmli buffer (1% SDS, 5% glycerol, 30 mM Tris-HCl pH 6.8) containing 8 M Urea to facilitate protein dissolution and denaturation. DC Protein Assay (BioRad, Marnes-La-Coquette, France) was used for protein determination, according to manufacturer’s instructions. BSA was used as standard and absorbance was measured at 595 nm, using a Multiskan FC photometer (Fisher Scientific, Illkirch, France).

Polyacrylamide gels were cast (10-15% acrylamide concentration, according to protein size) and loaded with 30 to 60 μg SDS-denatured protein per well. After separation, proteins were transferred onto a PVDF membrane (Polyvinylidene difluoride, Biorad, Marnes-La-Coquette, France). After being blocked with TBS-BSA 5%, membranes were incubated overnight at 4° C with primary antibodies, then 1 h at RT with HRP-conjugated secondary antibodies. Membranes were washed with 1X TBS-0.1% Tween20 solution, then revealed using Immobilon Western chemiluminescent HRP substrate (Millipore SAS, Molsheim, France) with G-box (Ozyme, Fisher, Bioblock Scientific, Illkrich, France). Measurement of band intensities were done with ImageJ software.

### Flow cytometry

After 24 h treatment with 40 μM chloroquine, pLKO cells were detached from dishes with Versene (Gibco, Thermo Fisher, Illkirch, France). Cells were rinsed with PBS and 1 μL of ViobilityTM (Macs Miltenyi Biotech, Bergisch Gladbach, Germany) was added to each experimental test. Cells were twice rinsed in PBS and fixed 10 min in 4% PFA. Cells were rinsed again with PBS and permeabilized 30 min at 4° C in Perm buffer III (BD Biosciences, Le Pont de Claix, France). Cells were incubated 30 min at 4° C with anti-Oct4-FITC-conjugated antibody (Biolegend, Ozyme) or anti-CD133-BV421-conjugated antibody (Biolegend, Ozyme) or anti-GFAP-APC-conjugated antibody (Miltenyi Biotec) or anti-DCX-PE- conjugated antibody (BD Biosciences). Marked cells were rinsed in PBS and analyzed with a Cytoflex flow cytometer (ThermoFisher) and the Flowlogic software TM (Macs Miltenyi Biotech).

### SdFFF cell sorting

### SdFFF device

The SdFFF separation device used in this study derived from previously described apparatuses [47] as schematized in Figure 3A. The apparatus was composed of two 880 x 47 x 2 mm polystyrene plates, separated by a Mylar® spacer in which the channel was carved. Channel dimensions were 788 × 12 × 0.175 mm with two 50 mm V-shaped ends. The measured total void volume (channel volume + connecting tubing + injection and detection device) was calculated after injection and retention time determination of a non-retained compound (0.10 g/L benzoic acid (Merck Millipore, Lyon, France) solution, UV detection at 254 nm). Sedimentation fields were calculated as previously described [48] and were expressed in gravity units (1 g = 980 cm/s^2^). Elution signals were recorded at 254 nm with a SPD-20AV UV/VIS Detector (Shimadzu, Champ sur Marne, France) and a NI9211 (10 mV input) acquisition device (National Instruments France, Nanterre, France) operated at 3 Hz and connected to a PC computer by the means of a laboratory-developed Visual Basic software (VB pro, Ver 6.0, Microsoft Corp.).

### SdFFF elution conditions for cell sorting and monitoring

The optimal elution conditions were determined experimentally and were: flow injection through the accumulation wall of 100 μL U87-MG, U87pLKO ± CQ 40 μM, U87shBeclin1 and U87shTrkC cell suspensions (2.5×10^6^ cells/mL); flow rate: 1.00 ± 0.02 mL/min; mobile phase: sterile PBS 1X (Gibco, Life Technologies, Cergy-Pontoise, France), pH = 7.4; external multi-gravitational field strength: 20.00 ± 0.02 g (365.5 ± 0.2 rpm). These conditions, optimal for cell separation [49] under the biocompatible hyperlayer elution mode [50], were essentially evidenced by i) the R_obs_ dependence (R_obs_ = void time /retention time : t_0_ / t_r_) to the flow rate and the external field strength, and ii) a value of the average cell elevation in the channel thickness "s", larger than the cell radius. By the use of the mean R_obs_ value, it was possible to calculate the average cell elevation "s" with the following equation: $R_{obs}=\frac{6s}{\omega }$ [51] where ω corresponds to the channel thickness (175 μm). These conditions were also implemented for monitoring the Time-dependent fractions, F, collected as described in Figure 3C to obtain the most pure fractions containing either more or less differentiated cells or cancer stem cells. The first fractions actually contain the largest and less dense cells, eluted after the the void peak and, at the end of the cell peak, the last fractions contain smaller and denser cells. To obtain a sufficient number of cells, consecutive SdFFF fractions were collected and gathered. Total Peak, TP corresponds to whole cell population eluted by SdFFF and is used as control for biological assays performed on sorted cells.

### Cell size analysis

A 256 channel Multisizer II Coulter Counter (Beckman Coulter, Fullerton, CA) was used to determine the mean diameter of the cell population. Cells were diluted in Isoton® (Beckman Coulter) to a final volume of 15 mL. The counting conditions were: 500 μL sample volume, cumulating three successive assays. Results were displayed as the mean ± standard deviation for three independent experiments.

### Statistical analysis

All experiments were performed at least 3 times or more. For small number of samples (less to 10) Kruskall Wallis analysis were done and for more than 10 samples ANOVA tests were performed. All analyses were realized using the PAST 2.17c software.

## Supplementary Material

Supplementary Figures
