# Supplementary material for: Autophagy inhibition reinforces stemness together with exit from dormancy of polydisperse glioblastoma stem cells
Source: Aging (Albany NY). 2021 Jul 27;13(14):18106–30. doi: 10.18632/aging.203362 (PMC8351723; doi:10.18632/aging.203362)
Supplement: Supplementary Figures [file aging-13-203362-s001.pdf]

SUPPLEMENTARY FIGURES

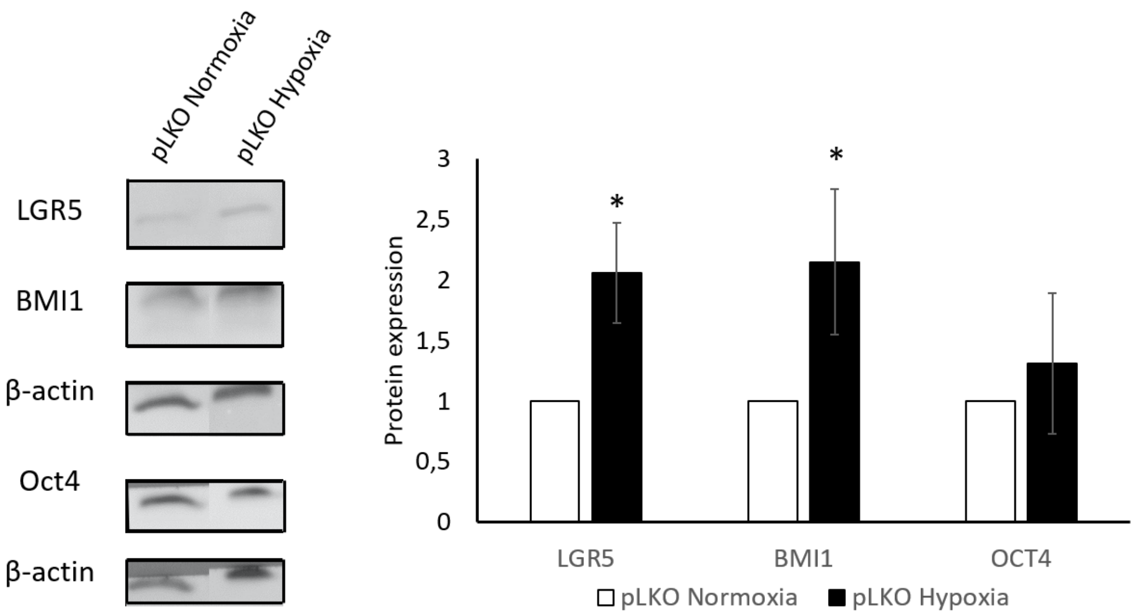

Supplementary Figure 1. Proteomic expression of CSCs markers and quantification in U87pLKO cell line cultured in normoxia and hypoxia. Cancer stem cells markers' expressions are enhanced when placed in hypoxia ; the difference is significant (*p<0.05) for LGR5 and BMI1 (N=3).

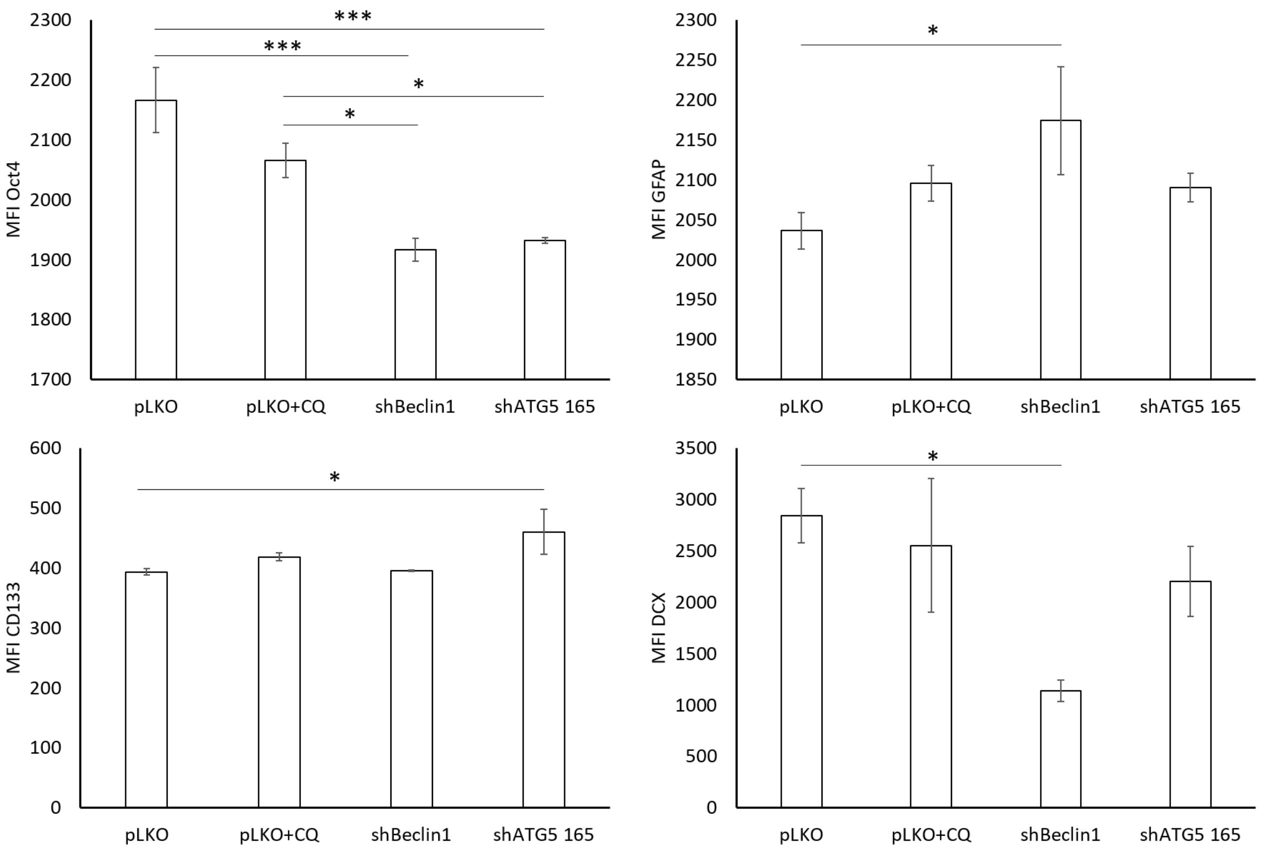

Supplementary Figure 2. MFI of Oct4, CD133, GFAP and DCX in U87 cell lines. Left panel : MFI of cancer stem cell markers. MFI of Oct4 significantly decreases after inhibition of autophagy. CD133 MFI significantly increases in U87shATG5 cells compared to basal condition *p<0,05. Right panel : MFI of GFAP significantly increases in U87shBeclin1 and DCX significantly decreases in the same cell line compared to pLKO (*p<0,05) (N=3).

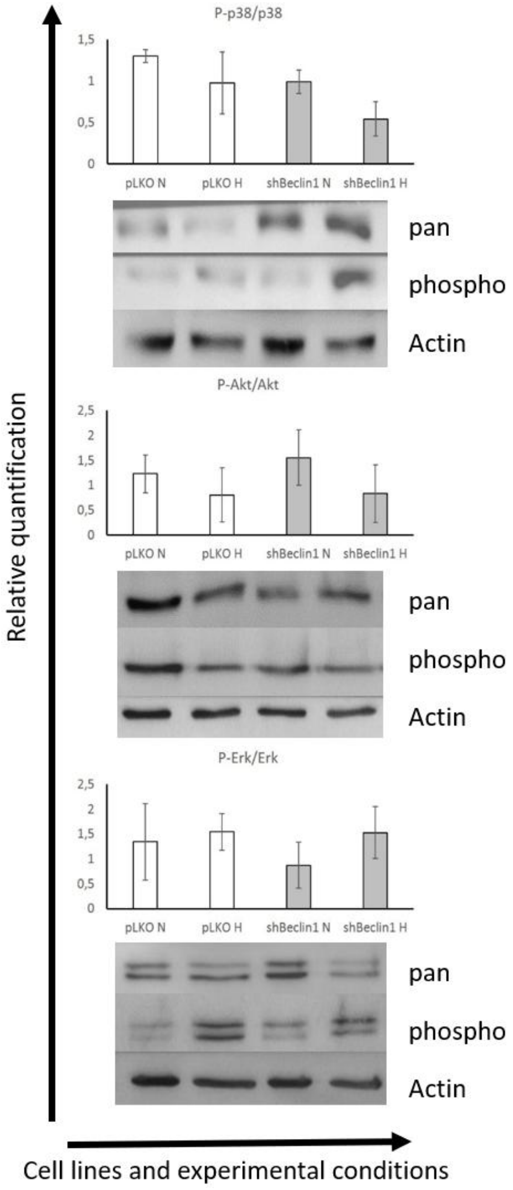

Supplementary Figure 3. Signaling pathway investigations. The p38, AKt and Erk signaling pathway activations are explored by western blot analysis. After 96h of culture in normoxia or hypoxia proteins from U87pLKO and U87shBeclin1 cells are analyzed. There is no difference of p38, AkT and Erk signaling pathway activation among both cell lines whatever cell the considered culture conditions (N=3).

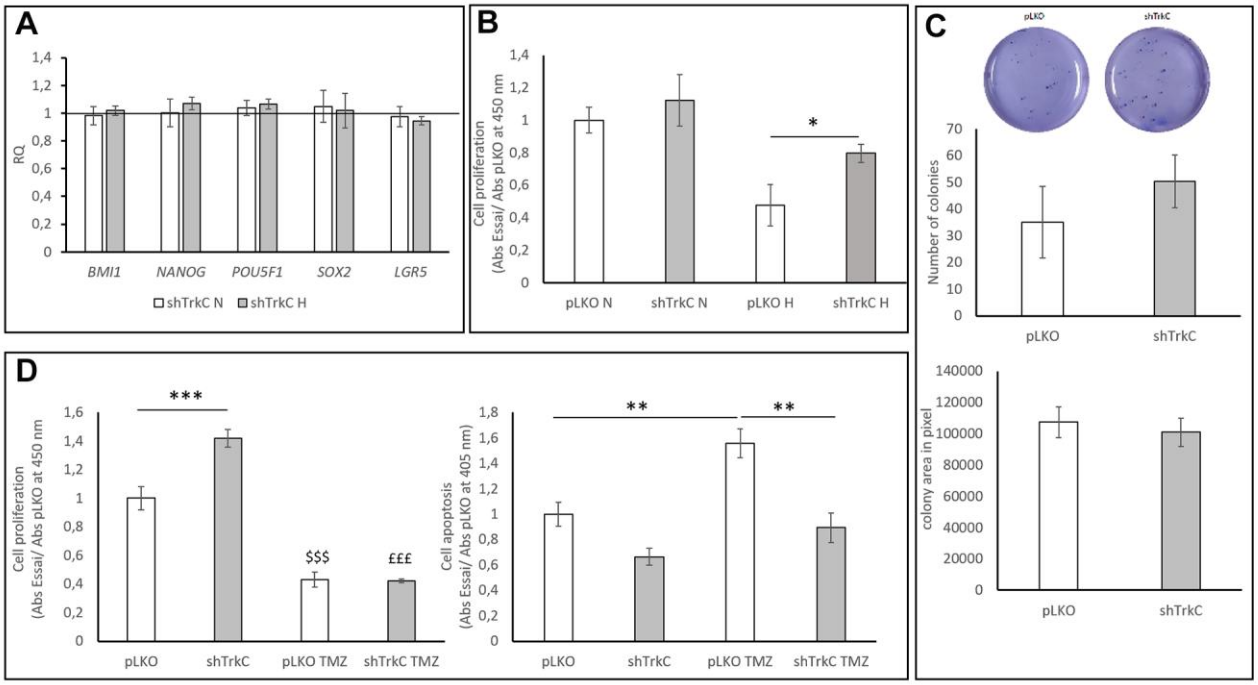

Supplementary Figure 4. (**A**) Cancer stem cells markers' expression in U87shTrKC cultured in normoxic (N) or hypoxic (H) condition. Only POU5F1 gene expression is significantly increased in U87shTrkC cultured in hypoxia compared to U87pLKO in the same culture condition * p<0.05. LGR5 gene expression is significantly decreased in U87shTrkC cultured in hypoxia compared to U87pLKO in the same experimental conditions * p<0.05 (N=4). (**B**) Cell proliferation After 3h of BrdU incorporation in hypoxic condition U87shTrkC occur to be significantly more proliferative than U87pLKO cells * p<0.05 (N=3). (**C**) Colony forming unit assay There is no difference in size or number of colonies form by U87pLKO and U87shTrkC cells (N=3). (**D**) TMZ impact on cell proliferation and cell apoptosis Left panel: In basal condition U87shTrkC proliferate significantly more than U87pLKO cells *** p<0.001. Temozolomide induced a significant decrease of cell proliferation in both U87pLKO and U87shTrkC cells compared to control (respectively $$$ p<0.001 for pLKO TMZ vs pLKO and £££ p<0.001 for shTrkC TMZ vs shTrkC) (N=3). Right panel: Temozolomide significantly increased apoptosis in U87pLKO cells compared to control ** p<0.01. Temozolomide failed to induce apoptosis in U87shTrkC cells leading to a significant decrease of cell apoptosis in U87shTrkC compared to U87pLKO when treated with temozolomide ** p<0.01 (N=3).

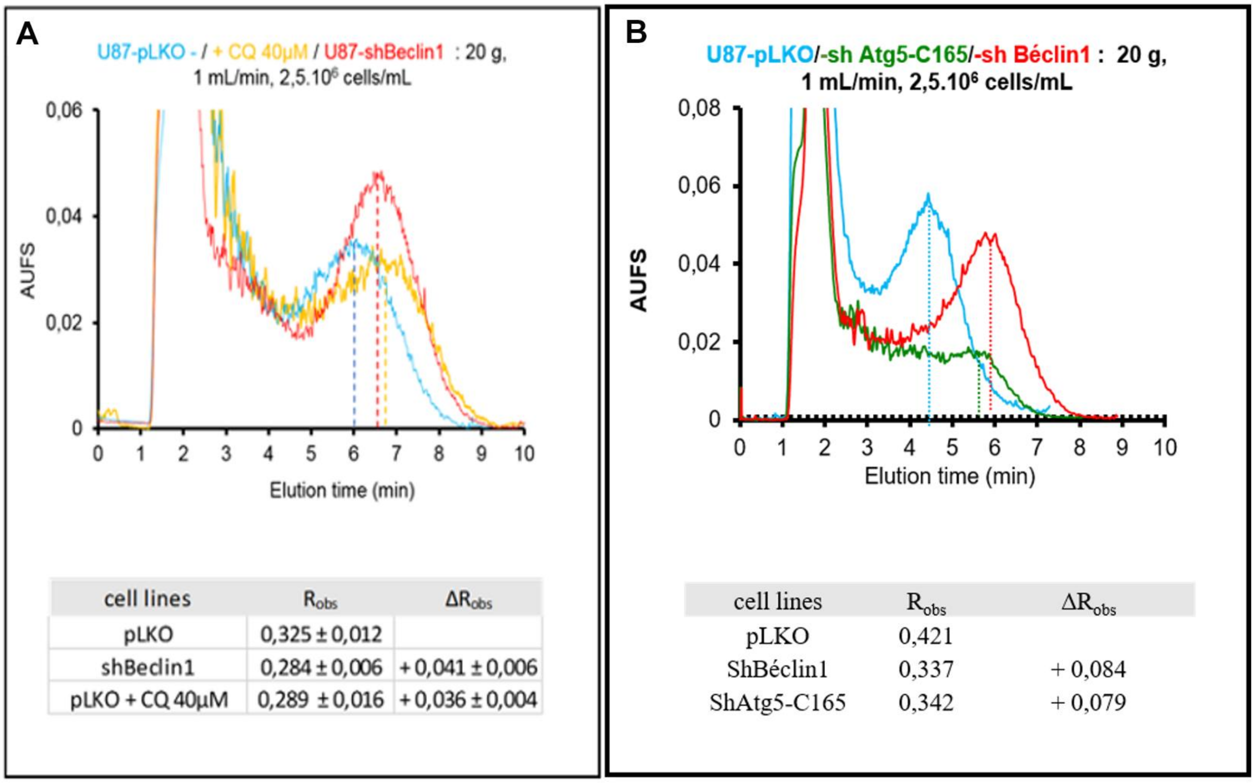

cell lines	R_{obs}	ΔR_{obs}
pLKO	0,325 ± 0,012	
shBeclin1	0,284 ± 0,006	+ 0,041 ± 0,006
pLKO + CQ 40µM	0,289 ± 0,016	+ 0,036 ± 0,004

cell lines	R_{obs}	ΔR_{obs}
pLKO	0,421	
ShBéclin1	0,337	+ 0,084
ShAtg5-C165	0,342	+ 0,079

Supplementary Figure 5. SdFFF monitoring of pharmacological (CQ) or genetic (shRNA) autophagy inhibition. (**A**) Similar right shifts were observed for CQ-treated U87pLKO (yellow curve) and U87shBeclin1 (red curve) as compared with U87pLKO without CQ. This shift was quantified by similar $\Delta_{Robs} = R_{obs\ pLKO} - R_{obs\ beclin1} = + 0.041 \pm 0.006$ and $\Delta R_{obs} = R_{obs\ pLKO} - R_{obs\ pLKO}$ + CQ 40µM = + 0.036 ± 0.004 for U87shBeclin1 and pLKO + 40 µM CQ, respectively (N=4). (**B**) Similar right shifts were observed for shATG5 (green curve) and U87shBeclin1 (red curve) as compared with U87pLKO. This shift was quantified by similar ΔRobs = $R_{obs\ pLKO} - R_{obs\ beclin1} = + 0.084$ and $\Delta R_{obs} = R_{obs\ pLKO} - R_{obs\ shAtg5} = + 0.079$ for U87shBeclin1 and U87shATG5, respectively (N=4).

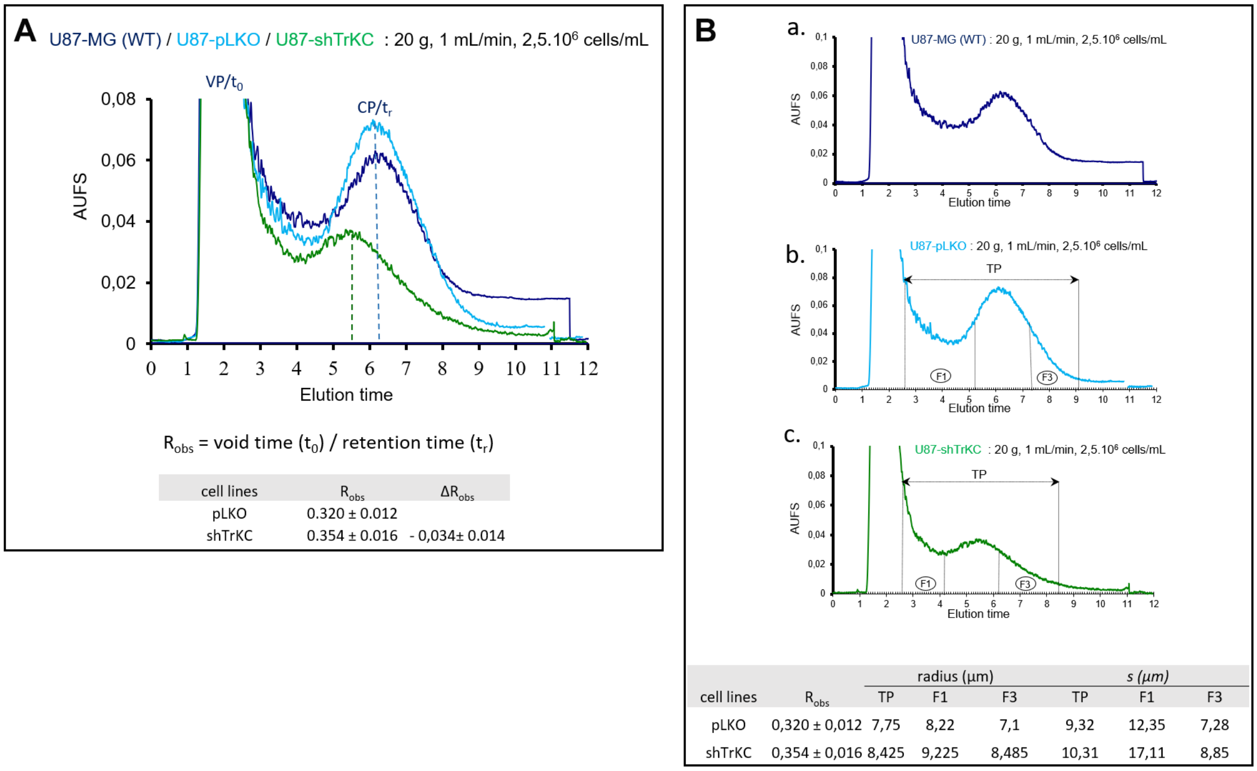

cell lines	R_{obs}	ΔR_{obs}
pLKO	0.320 ± 0.012	
shTrKC	0.354 ± 0.016	- 0,034± 0.014

cell lines	R_{obs}	radius (µm) TP	radius (µm) F1	radius (µm) F3	*s (µm)* TP	*s (µm)* F1	*s (µm)* F3
pLKO	0,320 ± 0,012	7,75	8,22	7,1	9,32	12,35	7,28
shTrKC	0,354 ± 0,016	8,425	9,225	8,485	10,31	17,11	8,85

Supplementary Figure 6. (**A**) Comparison of representative fractograms. In each case, classical two peak profiles were observed, the first peak corresponding to the void peak (unretained species) and the second peak corresponding to the cell peak. Elution profiles of U87-MG (dark blue curve) and pLKO (light blue curve) were similar, so U87pLKO was used as control for SdFFF cell sorting. We observed a left shift of U87shTrKC cell peak (green curve) as compared with U87pLKO. R_{obs} = t0 / tr (void time/retention time), calculated by the first moment method, were used for this peak shift measurement: $\Delta R_{obs} = R_{obs\ pLKO} - R_{obs\ shTrKC} = -0.034 \pm 0.013$. Cells collected in fraction 1 (F1) are larger than those eluted in the last one (F3). "s" considered as the average cell elevation in the channel thickness was larger than the particle radii of U87pLKO and U87shTrKC cells (N=4). (**B**) Representative fraction collections. Elution profiles of U87-MG (a) and U87pLKO (b) cells were similar, contrary to U87shTrKC cells (c). Fraction collections TP, F are indicated on fractograms by horizontal lines bounded by arrows and vertical plane lines respectively: TP: 2’30-9’00, F1: 2’30-5’10 and F3: 7’10-9’00 for U87pLKO; TP: 2’30-8’25, F1: 2’30-4’10 and F3: 6’10-8’25 for U87shTrKC (N=4).

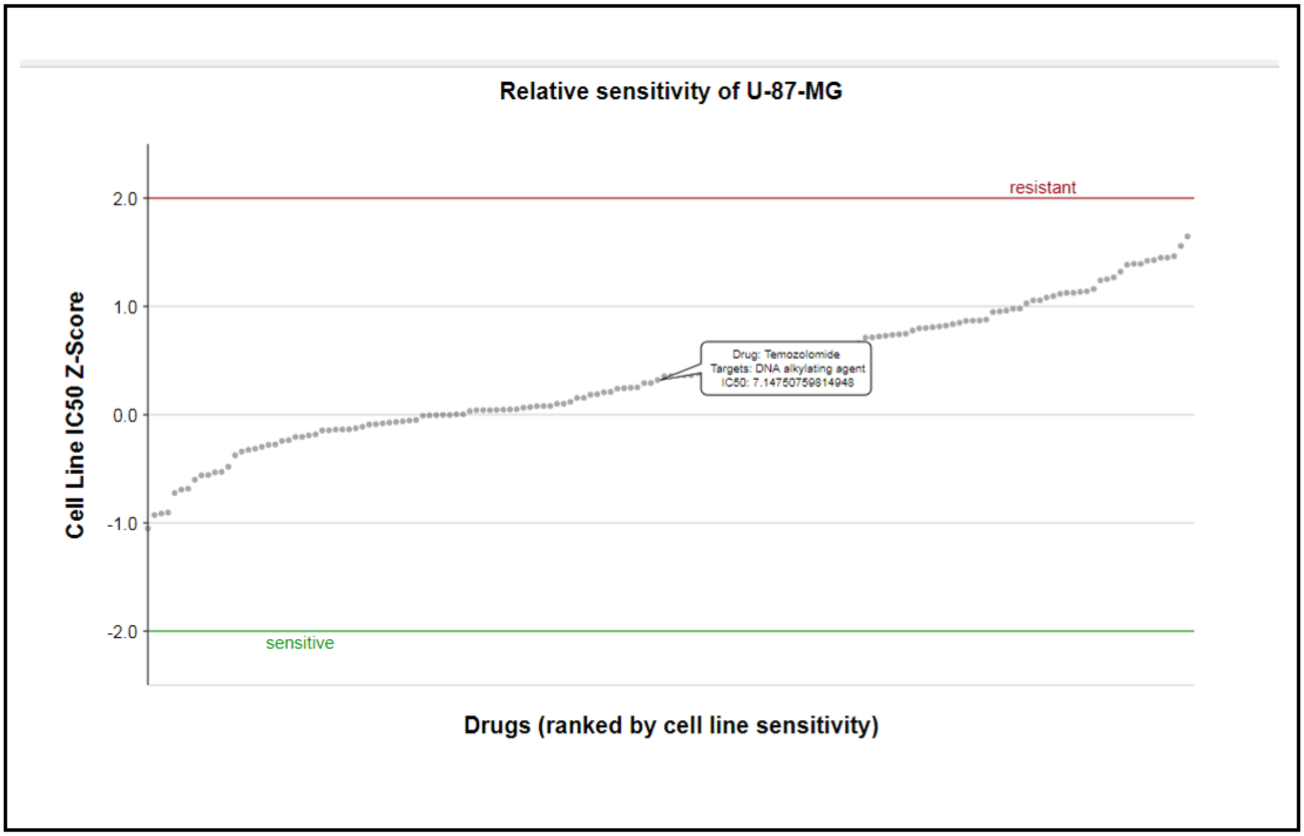

Supplementary Figure 7. U87-MG TMZ relative sensitivity. In U87-MG cells, IC50 Z-score of temozolomide is close to zero. U87-MG cells are considered resistant rather than sensitive to this drug [36].
